# mSphere of Influence: The complex world of bacterial biogeography

**DOI:** 10.1128/msphere.00628-23

**Published:** 2023-12-15

**Authors:** Andrew A. Bridges

**Affiliations:** 1 Department of Biological Sciences, Carnegie Mellon University, Pittsburgh, Pennsylvania, USA; University of Georgia, Athens, Georgia, USA

**Keywords:** biogeography, biofilm, community

## Abstract

Drew Bridges works in the field of bacterial signal transduction and studies the formation and disassembly of bacterial biofilms. In this mSphere of Influence article, he reflects on how the paper “Biogeography of a human oral microbiome at the micron scale” by Mark Welch et al. (J. L. Mark Welch, B. J. Rossetti, C. W. Rieken, F. E. Dewhirst, et al., Proc Natl Acad Sci U S A 113:E791–E800, 2016, https://doi.org/10.1073/pnas.1522149113) inspired him to change his research trajectory.

## COMMENTARY

Historically, bacteria have been thought of as comparatively simple organisms that lack the panache of their eukaryotic, “higher organism,” counterparts. We now know that this is not the case; bacteria exhibit complex behaviors, they analyze their surroundings to inform lifestyle decisions, they communicate, and they often form elaborate multi-species communities (1). As a graduate student studying cell biology, my eyes were opened to the complexity and beauty of bacterial communities through the work of Mark Welch et al. in their 2016 paper, “Biogeography of a human oral microbiome at the micron scale” (2). Their kaleidoscope-esque micrographs, which reflect the underlying physiology of an organized microbial system, inspired me to pursue a field change that led me on my current trajectory.

Biogeography is the study of where living things are found on Earth and why they are found there (3). Evolution patterns the spatial organization of living systems across size scales, from the global level to regional ecosystems to the micron scale, where microbes reign. As far back as Aristotle, naturalists have examined the spatial distribution of living organisms by eye, yet only with the advent of microscopes and molecular taxonomy has biogeography become accessible at the micron scale. Prior to the study discussed here, the same research group contributed a major technical advance to studies of biogeography at the micron scale (4). In this work, the authors developed combinatorial labeling and spectral imaging fluorescence *in situ* hybridization (CLASI-FISH) to differentiate dozens of taxa simultaneously in synthetic and natural mixtures of bacteria at high resolution. This approach enabled a systems-level analysis of microbial communities and their spatial interactions in unprecedented detail.

In the 2016 study, the authors focus their research on the relevant, accessible, and historically significant community of microbes found in dental plaque (2). Indeed, dental plaque was among the first samples visualized by van Leeuwenhoek through his rudimentary microscope (5). What the authors reveal is an exquisite organization that could not be described by preceding metagenomic studies (2). The group showcases a complex plaque consortia of nine predominant taxa formed around filamentous corynebacteria cells. The assemblies varied in size, spanning tens to hundreds of microns, with a reproducible spatial pattern. The spatial arrangement of each species within the configuration is suggestive of their underlying physiology, for example oxygen-sensitive species localized deep within the consortium, whereas those requiring or tolerating oxygen characteristically localized toward the community edge. Moreover, consumers of specific metabolites were found to be adjacent to producers. Collectively, this work provides a glimpse into the complex interspecies dynamics that sculpt the spatial arrangement of microbial communities.

Could the patterns of interaction and metabolic dependencies observed in dental plaque be mirrored in other microbial habitats, perhaps even pointing to universal principles of microbial organization? Current evidence suggests that microbial organization, division of labor, and specialization within natural communities are the norm (6–8). As the field journeys deeper into understanding how biogeography is established, it will be critical to use defined, multi-species, and genetically tractable communities to uncover mechanisms. Can spatial organization reminiscent of the communities observed in this study be reconstituted and monitored through development? Can genetic screens be developed to identify the critical molecular factors involved in spatial patterning? Moreover, what are the practical applications? Can we harness the knowledge of microbial biogeography to develop novel therapeutic strategies, influence microbial community behaviors, or even engineer our own microbial systems? The future of biogeography at the micron scale is rife with potential, and each answer will inevitably lead to even more questions.
